# Efficacy and safety of the commercial Chinese polyherbal preparation *Liu Shen Wan* as an adjunctive treatment for herpes zoster and postherpetic neuralgia: a systematic review and meta-analysis

**DOI:** 10.3389/fphar.2025.1698753

**Published:** 2025-11-28

**Authors:** Yajing Li, Le Zhang, Wenya Wang, Hui Zhao, Xing Liao

**Affiliations:** 1 Institute of Basic Research in Clinical Medicine, China Academy of Chinese Medical Sciences, Beijing, China; 2 China Center for Evidence-based Traditional Chinese Medicine, Beijing, China

**Keywords:** Liu Shen Wan, herpes zoster, postherpetic neuralgia, systematic review, randomized controlled trials

## Abstract

**Background:**

*Liu Shen Wan* (LSW), a commercial Chinese polyherbal preparation (CCPP), is frequently utilized as an adjuvant treatment for herpes zoster and postherpetic neuralgia (HZ and PHN). Nevertheless, the clinical efficacy and safety of this treatment remain uncertain.

**Purpose:**

This study aims to systematically evaluate the efficacy and safety of LSW as adjunctive treatment in treating HZ/PHN.

**Methods:**

A comprehensive search was conducted across PubMed, Web of Science, Embase, Cochrane Library, ClinicalTrials.gov, and four Chinese databases. Eligibility criteria (PICOS) included the following: (1) patients with HZ/PHN; (2) LSW as adjunctive therapy (experimental group); (3) conventional treatment only (control group); (4) primary outcomes: vesicle cessation, scab formation, VAS, and PHN incidence; secondary outcomes: scab shedding time, time to pain resolution, duration of pain persistence, PHN efficacy, and adverse reactions; and (5) RCTs. Risk of bias was assessed using ROB 2.0, and data synthesis/analysis used RevMan 5.4. No restrictions on language.

**Results:**

A total of 21 RCTs (n = 1,478) were included. Meta-analysis demonstrated that LSW plus conventional treatment significantly outperformed conventional treatment alone in shortening vesicle cessation time [MD = −1.44, 95% CI (−1.66, −0.93), *p* < 0.00001, I^2^ = 70%], accelerating scab formation (MD = −1.72, 95% CI (−2.09, −1.35), *p* < 0.00001, I^2^ = 38%), reducing scab shedding time (MD = −2.22, 95% CI (−3.64, −0.80), *p* = 0.002, I^2^ = 36%), decreasing time to pain resolution (MD = −2.46, 95% CI (−3.52, −1.39), p < 0.00001, I^2^ = 0%), and shortening pain persistence duration (MD = −1.97, 95% CI (−2.49, −1.46), *p* < 0.00001, I^2^ = 86%). Additionally, the combination therapy reduced PHN incidence (RR = 0.24, 95% CI (0.10, 0.57), *p* = 0.001, I^2^ = 0%), improved PHN efficacy (OR = 6.11, 95% CI (2.91, 12.82), *p* < 0.00001, I^2^ = 61%), and lowered adverse reactions (RR = 0.60, 95% CI (0.37, 0.96), *p* = 0.03, I^2^ = 0%). No serious drug-related adverse events were reported.

**Conclusion:**

Adjunctive LSW therapy demonstrates potential to shorten herpes lesion healing time, improve treatment outcomes, and effectively prevent postherpetic neuralgia compared to conventional treatment alone. It also significantly reduces both the duration of pain and the overall disease course. Nevertheless, limitations in the current evidence base, including study quality and quantity, necessitate further rigorous investigation to confirm the long-term efficacy and safety profile of this combined intervention.

**Systematic Review Registration:**

https://www.crd.york.ac.uk/PROSPERO/view/, identifier CRD42024595203.

## 1 Introduction

Herpes zoster (HZ), often referred to as shingles, is an acute viral infection caused by the reactivation of the varicella-zoster virus (VZV) and is characterized by skin lesions and neuropathic pain (Werner et al., 2017; Consensus Workgroup On Herpes Zoster and Diseases, 2022). Globally, the condition has an incidence rate of 3–5 per 1,000 person-years in North America, Europe, and the Asia-Pacific, typically manifesting as a painful vesicular rash with dermatomal distribution (Kawai et al., 2014). A community-based retrospective survey focusing on HZ among Chinese individuals aged ≥50 years documented a cumulative incidence of 22.6 cases per 1,000 population, with marked geographical disparities between rural (17.2/1,000) and urban (39.5/1,000) cohorts (Li et al., 2016). The annualized incidence rate of 3.43 per 1,000 person-years (rural: 2.06; urban: 7.65) demonstrated consistency with both regional observations in Guangdong, China (Zhu et al., 2015) and multinational epidemiological benchmarks of 3–5 cases per 1,000 population across North America, Europe, and the Asia-Pacific region (Kawai et al., 2014). As the most frequent neurological complication of HZ (Jiang et al., 2023), postherpetic neuralgia (PHN) is a chronic neuropathic pain syndrome persisting ≥3 months after HZ rash resolution (Hadley et al., 2016). Although aspirin and mild analgesics are empirically prescribed for PHN, their efficacy remains suboptimal due to the inherent opioid-resistant nature of neuropathic pain (Kost and Straus, 1996). Recent studies have explored the efficacy of other traditional Chinese medicine (TCM) therapies, such as acupuncture and moxibustion, in managing PHN and its associated pain, demonstrating promising results and highlighting the broader therapeutic value of traditional approaches in this field (Wu et al., 2021; Hu et al., 2021).

*Liu Shen Wan* (LSW), a TCM formulation which can quickly detoxify swelling, clear away heat, and relieve pain, has been widely used for over a century in the management of influenza, tonsillitis, pharyngitis, and mumps (Ma et al., 2020). Recent advances in understanding the modern pharmacological effects and clinical applications of LSW have revealed expanded therapeutic potential. Research demonstrates its efficacy not only in symptomatically relieving pharyngeal disorders (Wang et al., 2021) but also in treating HZ and PHN (Ren T, 1998; Feng and Chen, 2008). This TCM formula, pharmacologically characterized by antipyretic, detoxifying, anti-inflammatory, and analgesic properties, is highlighted as a potential adjunctive therapy with antiviral and analgesic effects in both acute-phase HZ and PHN. LSW is a CCPP composed of six botanical drugs: *Calculus bovis* (Bovidae; the desiccated gallstone of *Bos taurus* domesticus Gmelin), *Moschus moschiferus* (Cervidae; preputial gland secretion of musk deer), *Venenum bufonis* (Bufonidae; dried secretion of *Bufo bufo* gargarizans or *Bufo melanostitus*), Pernulo (Pteriidae; Pearl), realgar (As_4_S_4_) (mineral; realgar ore), and *Borneolum syntheticum* (Lauraceae; fresh twigs and leaves) (Zhao et al., 2021) (Table 1). All constituents are listed in the 2020 edition of the *Chinese Pharmacopoeia*. Existing evidence shows that studies on the effectiveness of LSW as adjunctive treatment in treating HZ and PHN are not consistent (Yin and Liang, 2006; Zhan et al., 2008; Qian and Lu, 2017). This inconsistency necessitates meta-analysis for definitive efficacy assessment.

**TABLE 1:** Composition of commercial Chinese polyherbal preparation *Liu Shen Wan*.

Scientific name	Latin name	Herb name in Chinese	Family	Parts used	Reporting in original studies (n)	
					Methods and discussions	Discussions
Bezoar	*Calculus bovis*	Niuhuang	Bovidae	Gallstone	2	13
Musk	*Moschus moschiferus*	Shexiang	Cervidae	Preputial gland secretion		
Toad venom	*Venenum bufonis*	Chansu	Bufonidae	Dried secretion		
Pearl	Pernulo	Zhenzhu	Pteriidae	Pearl		
Realgar	Realgar	Xionghuang	Mineral	Realgar ore		
Borneol	Borneolum syntheticum	Bingpian	Lauraceae	Fresh twigs and leaves		

This systematic review aims to quantify therapeutic outcomes through the meta-analysis of randomized controlled trials (RCTs) to establish statistically robust conclusions. This study seeks to evaluate the effectiveness and safety of LSW, including the incidence of HZ and PHN, the occurrence of adverse reactions, the period to cessation of shingles rash, time to crusting, scab detachment, time to onset of pain relief, and time to pain resolution.

## 2 Materials and methods

Preferred Reporting Items for Systematic Reviews and Meta-Analyses Protocols, PRISMA-P 2015 (Moher et al., 2015) was completed and registered with PROSPERO (registration number: CRD42024595203).

### 2.1 Standardization and taxonomic validation of the composition of Liu Shen Wan

The scientific names of all drug components were standardized based on the 2020 edition of the *Chinese Pharmacopoeia* (https://ydz.chp.org.cn/#/main). Concurrently, all corresponding plant species were taxonomically validated using Medicinal Plant Names Services (MPNS) (http://mpns.kew.org/mpns-portal/) or Plants of the World Online (http://www.plantsoftheworldonline.org). Full species names, including authorities and family, have been included, along with the drug name. Summary tables describing the composition of the ingredients and how they were reported in the original study are presented in Table 1. There were 13 studies that mentioned composition in their discussion, while two detailed it in both their methods and discussion.

### 2.2 Search strategy

A comprehensive literature search was performed across multiple international databases, including PubMed, Web of Science, Embase, Cochrane Library, ClinicalTrials.gov, and four additional Chinese electronic databases using keywords such as “*Liu Shen Wan*” “herpes zoster,” “postherpetic neuralgia”, and “randomized controlled trials” from the establishment of the database to 6 April 2025. We performed a logical search by combining subject terms and free terms using Boolean operators (Supplementary Material A). To minimize regional bias, the search was performed without any restrictions on language and publication type. The literature search was conducted without applying any database filters.

### 2.3 Criteria for study inclusion in this review

The eligibility criteria were formulated based on the PICOS (Population, Intervention, Comparison, Outcomes, and Study) framework, which is a standard approach for defining inclusion/exclusion criteria in systematic reviews (Amir-Behghadami and Janati, 2020; Richardson et al., 1995).

Inclusion criteria: (1) patients with HZ/PHN; (2) LSW as adjunctive therapy (experimental group); (3) conventional treatment only (control group); (4) primary outcomes: vesicle cessation, scab formation, VAS, and PHN incidence; secondary outcomes: scab shedding time, time to pain resolution, duration of pain persistence, PHN efficacy, and adverse reactions; and (5) RCTs.

Exclusion criteria: duplicated publications; research without full text, studies with missing data for extraction; animal studies or non-original research; studies with unavailable full text or insufficient data for extraction; and studies failing to measure or report outcomes of interest.

### 2.4 Quality assessment

The quality of the included studies was assessed using the Cochrane collaboration tool for assessing risk of bias in randomized controlled trials (RoB 2.0) (Sterne et al., 2019). Two review authors (LYJ and ZL) independently assessed potential risks of bias in a single result using the Cochrane tool in all five domains (bias arising from the randomization process; bias due to deviations from intended interventions; bias due to missing outcome data; bias in measurement of the outcome; and bias in selection of the reported result) (Higgins, 2024) in each study. The overall risk of bias for each included trial was categorized as follows. “Low risk”: trials with low risk of bias across all assessed domains. “Some concerns”: trials with concerns in ≥1 domain, but without high risk in any domain. “High risk”: trials with high risk in ≥1 domain, or multiple concerns substantially compromising result reliability. This classification aligns with the Cochrane RoB 2.0 framework, ensuring the standardized evaluation of randomization, deviations, missing data, measurement, and reporting biases (Boutron, 2024). The discrepancy in judgment was resolved by another author (LX or ZH).

### 2.5 Selection of studies and data extraction

The literature search was systematically conducted using NoteExpress reference management software, followed by implementation of automated deduplication algorithms to eliminate redundant records. Subsequently, two reviewers (LYJ and ZL) involved in the study independently conducted a rigorous screening based on predefined inclusion and exclusion criteria. The initial screening phase was to exclude irrelevant studies based on the titles and abstracts, followed by a full-text review of the remaining studies to ensure they met the requirements for the meta-analysis. Data were extracted from eligible studies, including patient demographics, study design, intervention details, efficacy outcomes (e.g., pain scores and rash improvement), and safety data. Inter-rater disagreements were adjudicated by a senior investigator (LX or ZH).

### 2.6 Data analysis

Data synthesis and statistical analysis were performed using RevMan 5.4 software. For dichotomous data, risk ratio (RR) or odds ratio (OR) were used to represent the effect statistic, while for continuous data, mean difference (MD) or standardized mean difference (SMD) were appropriate measures of effect size, with 95% confidence intervals (CIs) (Higgins Jpt, 2024). Given anticipated heterogeneity across studies due to variations in population characteristics and intervention effects, this study employed random-effects meta-analysis models for all quantitative syntheses. Sensitivity analysis was conducted by removing one study at a time. If the results were unstable, this study considered removing specific studies and performing descriptive analyses or further subgroup analyses.

The degree of heterogeneity was calculated in the Q test and quantified by the I^2^ statistic. Chapter 10 in the latest *Cochrane Handbook* guidelines was followed for interpreting heterogeneity (Jj et al., 2024): I^2^ less than 40% indicated minimal heterogeneity, 30%–60% was considered moderate heterogeneity, 50%–90% suggested substantial heterogeneity, and I^2^ greater than 75% denoted considerable heterogeneity. The importance of the observed I^2^ value depends on both the magnitude and direction of effects and the strength of evidence for heterogeneity, which can be assessed through measures such as the *p*-value from the Chi^2^ test or I^2^ confidence intervals, though its reliability decreases with fewer studies. When heterogeneity was significant, subgroup analysis was performed to explore potential sources of heterogeneity. Because it was suspected that the studies on the effectiveness of LSW as adjunctive treatment in treating HZ and PHN were not consistent, random effects models were conducted. For specific outcome measures, funnel plots were used to investigate publication bias when more than ten studies were included.

### 2.7 Evidence quality assessment

In this systematic review and meta-analysis, the Grades of Recommendations Assessment Development and Evaluation (GRADE) framework was applied to evaluate the certainty of evidence for all critical outcomes. The evidence quality assessment followed the Cochrane methodology and encompassed five key domains: risk of bias, inconsistency, indirectness, imprecision, and publication bias (Balshem et al., 2011; Guyatt G. et al., 2011; Guyatt et al., 2011a; Guyatt et al., 2011b; Guyatt et al., 2011c). The certainty of evidence was categorized into four levels (high, moderate, low, or very low) for each outcome in our summary of findings tables, which present relative/absolute effect estimates alongside quality assessments (Schünemann, 2024). These tables were developed using GRADEpro GDT (https://gdt.gradepro.org/app/) (Prime, 2025) to ensure standardized reporting format compliance.

### 2.8 Assessment of consensus-based reporting guidelines for phytochemical characterization of medicinal plant extracts

To ensure methodological reproducibility and transparency in reporting of research on CCPP LSW, we adhered to the consensus-based reporting guidelines for phytochemical characterization of medicinal plant extract (ConPhyMP) statement and its online tool (https://ga-online.org/best-practice/) for reporting the composition and processing of the preparation (Heinrich and Jalil, 2023; Heinrich et al., 2022). Two authors (LYJ and ZL) independently assessed the included studies, guided by the ConPhyMP checklist, to evaluate the reporting quality of these studies (Heinrich et al., 2022). This process involved completing both Table 1 (general plant material) and Table 2 (pharmacopeial-recorded preparations), with the full checklists provided in Supplementary Material C. To resolve any discrepancies in evaluation, a consensus was sought through discussion or by consulting a third reviewer (LX).

**TABLE 2:** Characteristics and intervention details of the included trials.

Study ID	Treatment duration, follow-up duration	Duration of condition (mean or range)	No. of participants randomized/assessed	Age	Intervention	Comparator	Outcomes
Zhang (1998)	NS, NS	Total: 2–4 days	I: 35/35 C: 26/26	I: 35 C: 37	LSW + ribavirin + cimetidine	Moroxydine + VB_1_+VB_12_ + 0.1% crystal violet	②⑤
Zhang (1999)	7/14 days, 5, 8, 27 days	I: 2–8 days C: 2–9 days	I: 43/43 C: 41/41	I: 38.4 C: 36.5	LSW + moroxydine	Acyclovir + crystal violet	①③⑤⑥⑧
Lu (2001)	7 days, NS	Total: 1–4 days	I: 30/30 C: 30/30	Total: 14–56	LSW + poly (I:C)	Moroxydine + VB_1_	⑤
Qiu (2002)	7 days, 3, 7, 14, 21, 28 days	I: 2.5 C: 2.4	I: 28/28 C: 28/28	I: 41 C: 42	LSW + ribavirin +5% GS + ibuprofen +VB_1_ +VB_12_ +VE	Ribavirin + 5% GS +ibuprofen + VB_1_ +VB_12_ +VE	②③④⑤
He (2002)	NS, NS	I: 3–5 C: NS	I: 28/28 C: 20/20	I: 15–68 C: 13–69	LSW + poly (I:C)	Moroxydine + VB_1_	⑤
Zheng (2004)	NS, NS	Total: <2 days	I: 23/23 C: 25/25	Total: 19–50	LSW + cimetidine	Moroxydine + VB_1_ + VB_12_	①②⑤⑧
Liu (2004)	7 days, NS	Total: 3 days	I: 21/21 C: 17/17	I: 43 C: 42	LSW + acyclovir + VB_12_	Poly (I:C) + VB_12_	①②⑤
Fan (2005)	NS, NS	I: 3–5 C: 2–5	I: 14/14 C: 14/14	I: 18–64 C: 17–65	LSW + poly (I:C)	Moroxydine/antiviral drug + VB_1_	⑤
Wang (2005)	10 days, NS	Total: 3 days	I: 46/46 C: 42/42	I: 47 C: 45	LSW + PDS	Hepatunn	①②⑤
Yin and Liang (2006)	NS	Total: 1–3 days	I: 21/21 C: 19/19	Total: 20–58	LSW + acyclovir + VB_12_	Acyclovir + VB_12_	②⑤
Zhan et al. (2008)	NS, 7–10 days	I: 1–4 days C: 1–5 days	I: 28/28 C: 20/20	I: 17–63 C: 18–60	LSW + 5% GS + ribavirin	Calamine lotion + 5% GS + ribavirin	⑤
Yao and Sun (2011)	7 days, 7, 14, 21, 28 days	Total: <3 days	I: 13/13 C: 13/13	I: 45 C: 43	LSW + acyclovir	Acyclovir + ibuprofen	②③⑤④⑥⑧
Liu and Yang (2012)	14 days, NS	I: 1.5–7.0 months C: 1.3–6.8 months	I: 44/44 C: 30/30	I: 45–68 C: 43–67	LSW + 2% lidocaine + prednisolone acetate	Mecobalamin + acyclovir + VB_1_	⑧
Zeng et al. (2012)	14 days, NS	I: 1.9 ± 0.73 C: 2.0 ± 0.77	I: 61/61 C: 61/61	I: 65 ± 5.4 C: 66 ± 4.3	LSW + acyclovir + fursultiamine tablets + BCG-PSN	Acyclovir +fursultiamine tablets + calamine lotion	①②⑤⑥⑧
Li et al. (2013)	NS, NS	Total: ≤5 days	I: 91/91 C: 90/90	Total: 55.4	LSW + ganciclovir	Ganciclovir	①②⑤⑧
Hao (2014)	7 days, NS	NS	I: 42/42 C: 42/42	I: 31 C: 30	LSW + ganciclovir	Ganciclovir	①②⑤⑧
Zhang (2014)	7 days, NS	NS	I: 42/42 C: 42/42	I: 19–43 C: 18–45	LSW + ganciclovir	Ganciclovir	①②⑤⑥⑦⑧
Zhu (2014)	14 days, 14 days	I: 5.1 ± 1.0 C: 4.9 ± 1.8	I: 30/30 C: 30/30	I: 69.8 ± 11.8 C: 66.2 ± 10.4	LSW + DS	DS + acyclovir + acyclovir ointment	①②⑤⑥
Dong et al. (2015)	7 days, NS	NS	I: 40/40 C: 40/40	Total: 38.5	LSW + antiviral drug	Antiviral drug	②④
Zhang (2015)	NS, NS	NS	I: 24/24 C: 20/20	I: 21–82 C: 20–78	LSW + gabapentin capsules	Carbamazepine	⑧⑨
Qian and Lu (2017)	10 days, NS	I: 3.0 C: 2.5	I: 62/62 C: 62/62	I: 72 ± 11.8 C: 70 ± 10.6	LSW + acyclovir	Acyclovir	⑦

NS, not specified; C, control group; I, intervention group; PDS, potassium dehydroandrographolide succinate for injection; BCG-PSN, BCG, polysaccharide and nucleic acid preparation; SPSS, stroke-physiological saline solution; VB, vitamin B; Poly(I:C), polyinosinic–polycytidylic acid injection; DS, diclofenac sodium; GS, glucose injection; ① vesicle cessation time; ② time to scab formation; ③ scab shedding time; ④ time to pain resolution; ⑤ duration of pain persistence; ⑥ incidence of PHN; ⑦ efficacy of PHN; ⑧ adverse events; ⑨ visual analogue scale (VAS).

## 3 Results

**FIGURE 1:**
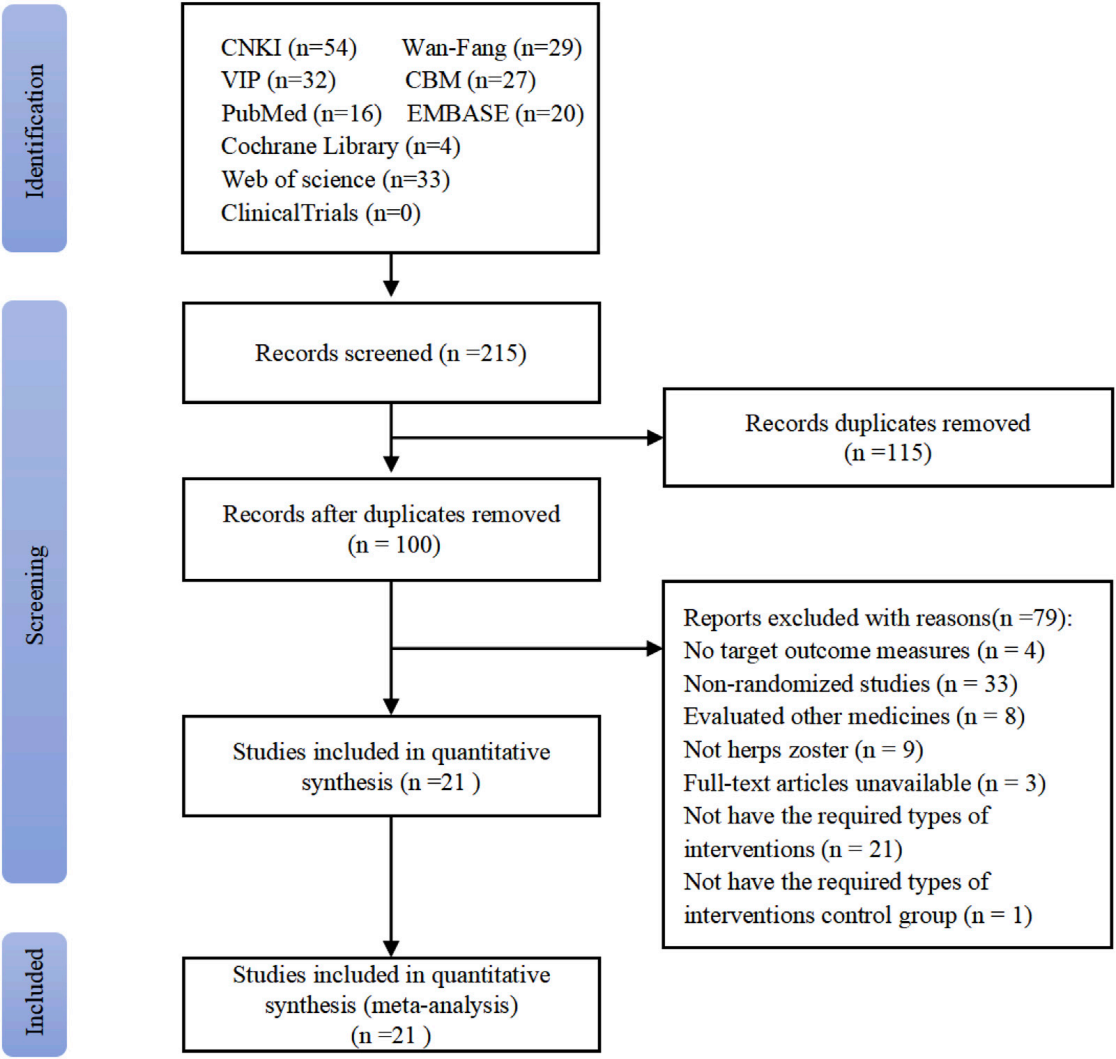
Study selection flowchart.

The systematic search yielded 215 initial records from target databases (Figure 1). After screening, 115 duplicates were excluded, and the full texts of 100 studies were reviewed. Included were 21 RCTs, with a total sample size of 1,478 participants (Zhang, 1998; Zhang, 1999; Lu, 2001; He, 2002; Qiu, 2002; Liu, 2004; Zheng, 2004; Wang C C, 2005; Fan, 2005; Yin and Liang, 2006; Yao and Sun, 2011; Liu and Yang, 2012; Hx, 2012; Zeng et al., 2012; Li et al., 2013; Hao, 2014; Zhu, 2014; Zhang, 2014; Dong et al., 2015; Zhang et al., 2015; Qian and Lu, 2017; Zhan et al., 2008). Key methodological characteristics, including study design, intervention protocols (e.g., LSW dosage forms and treatment duration), and control group comparators, are summarized in Table 2.

### 3.1 Characteristics of included studies

All 21 RCTs were conducted in China and compared the efficacy of LSW in conjunction with other medications versus the use of conventional treatment alone (Table 1). The treatment durations varied across studies, with the majority (seven trials) adopting a 7-day regimen, followed by 10-day (two trials) and 14-day (three) protocols. Notably, one trial implemented a flexible duration of either 7 or 14 days based on clinical severity, while the remaining trials did not explicitly specify their intervention periods. The follow-up designs exhibited notable heterogeneity across the trials: one study conducted weekly evaluations at 7, 14, 21, and 28 days; another implemented assessments at 3, 7, 14, 21, and 28 days; one study performed a single follow-up at 14 days; one adopted short-term follow-up of 7–10 days; and one utilized nonstandard intervals at 5, 8, and 27 days. The remaining trials did not explicitly specify their follow-up duration. The duration of HZ ranged from 1 to 9 days, with only three studies beyond 5 days.

The number of participants in the included studies varied from 26 to 181, and no studies lost participants to follow up. The included studies enrolled participants of a broad age spectrum (range: 9–83 years) (Wang, 2005), showing heterogeneous age distributions—whereas most cohorts had mean ages of 30–50 years, specific trials included older populations (mean age up to 72 ± 11.8 years in intervention groups) (Qian and Lu, 2017).

### 3.2 Intervention and comparator

All included studies assessed the efficacy of LSW as adjunctive treatment versus conventional treatment alone, with intervention protocols emphasizing integrative strategies. This combinatorial approach represents a divergence from conventional monotherapy paradigms, underscoring the potential for botanical drug synergy in symptom-targeted management. LSW was administered in three modes: oral (2 studies), external (7 studies), or oral plus external (12 studies). Synergies included combining LSW with antivirals (14 studies), antihistamines (1 study), TCM injections (1 study), antiepileptic drug (1 studies), or immunomodulators (4 studies). Comparator groups received guideline-recommended antiviral therapy (18 studies), antivirals (2 studies), and anticonvulsants (1 study) with adjunctive symptomatic management of systemic interventions (vitamin B1/B12 injections) and topical therapies (gentian violet, acyclovir ointment, or calamine lotion).

### 3.3 Risk of bias assessment

**FIGURE 2:**
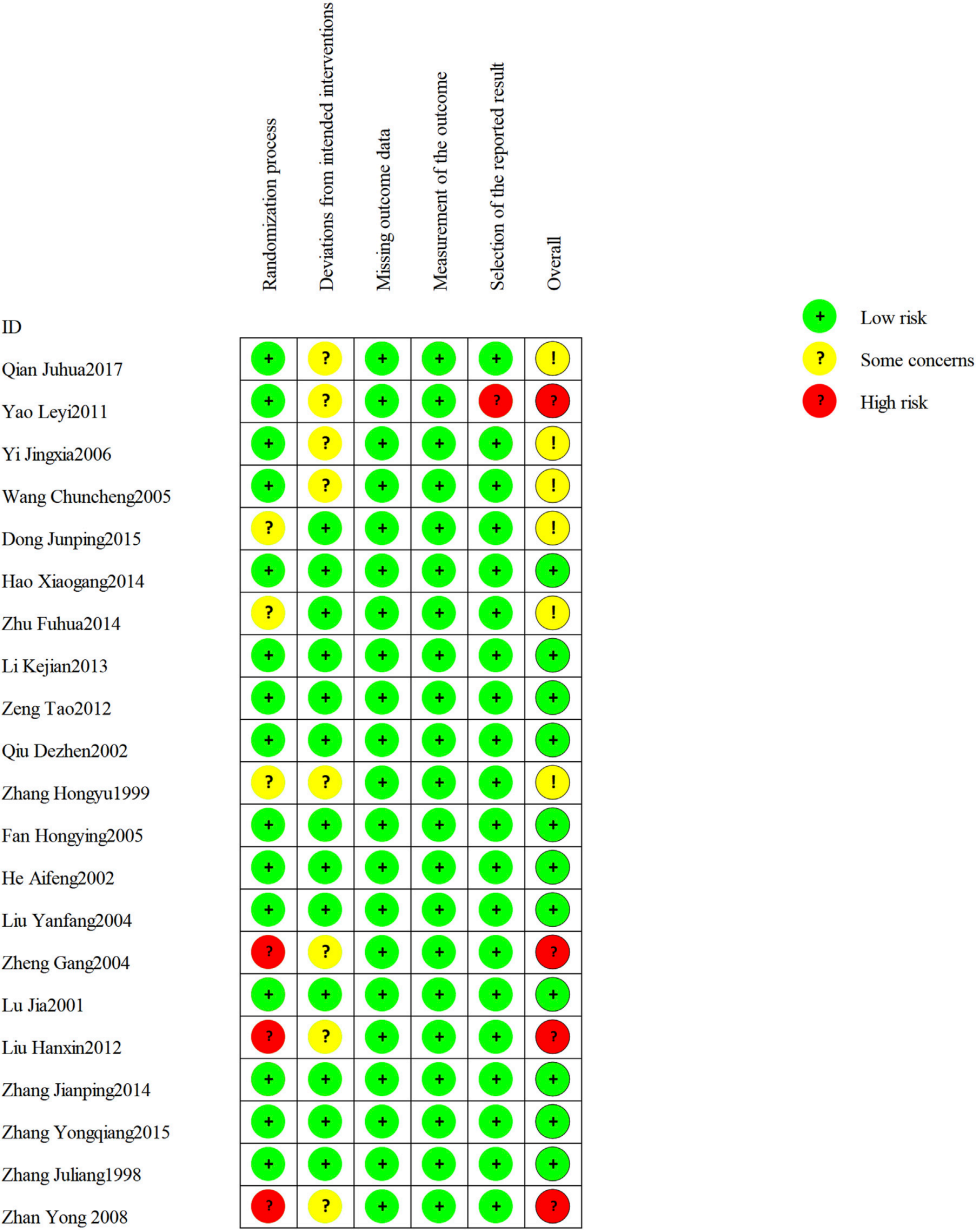
Risk of bias summary: review authors’ judgments about each risk of bias item for each included study.

**FIGURE 3:**
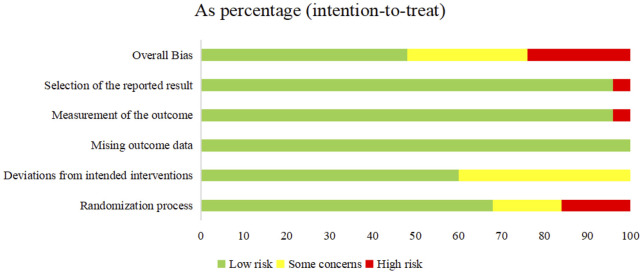
Risk of bias graph: review authors’ judgments about each risk of bias item presented as percentages across all included studies.

The 21 included RCTs were assessed according to the five domains of the risk of bias assessment tool RoB 2.0. The comprehensive evaluation outcomes are graphically summarized in Figures 2, 3. Risk of bias assessment revealed the following distribution: high risk in four RCTs, unclear risk in six RCTs, and low risk in the remaining 11 RCTs.

Among the 21 RCTs, one study (Zheng, 2004) was judged as high risk of bias for random sequence generation due to the use of an inappropriate randomization method. Two additional RCTs (Hx, 2012; Zhan et al. 2008) were judged to be at high risk of selection bias due to poor documentation on whether allocation concealment was maintained until participants were formally enrolled and assigned to their respective intervention groups. Three RCTs did not report any randomization details and were judged as unclear risk. For all included trials, the risk of attrition bias was low, and no missing data were reported. Eight trials (Zhang, 1999; Zheng, 2004; Wang, 2005; Yin and Liang, 2006; Zhan et al., 2008; Yao and Sun, 2011; Qian and Lu, 2017; Hx, 2012) met Cochrane RoB 2.0 criteria for risk of “some concerns” due to missing details on blinding implementation. One study (Yao and Sun, 2011) was considered as high risk of bias for selective outcome reporting.

### 3.4 Outcome

#### 3.4.1 Primary outcomes

##### 3.4.1.1 Vesicle cessation time

**FIGURE 4:**
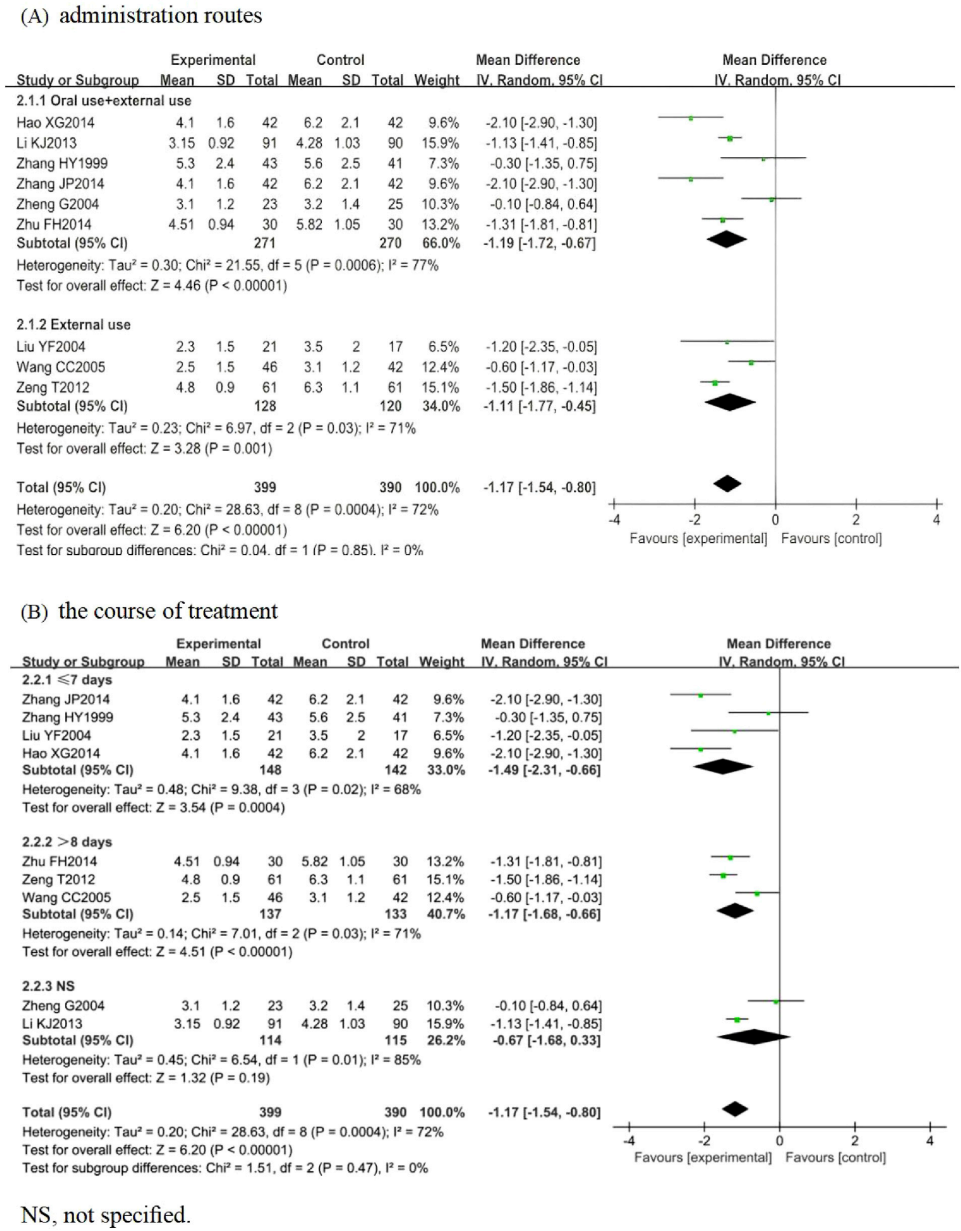
Forest plots with the random-effects model for vesicle cessation time. **(A)** Administration routes. **(B)** Course of treatment.

Nine RCTs involving 789 participants were included, among which 8 (n = 705) (Zhang, 1999; Liu, 2004; Zheng, 2004; Wang, 2005; Li et al., 2013; Hao, 2014; Zhu, 2014; Zeng et al., 2012) focused on HZ, while Zhang (2014) investigated PHN (n = 84). The pooled analysis indicated that the LSW plus conventional treatment group had a statistically significant shorter time to blister resolution than the control group (MD = −1.17, 95% CI (−1.54, −0.80), *p* < 0.00001, I^2^ = 72%) (Figure 4). Sensitivity analysis was conducted on these nine studies, and none interfered with the results of this meta-analysis, confirming that the pooled results were robust. Subgroup analysis was conducted regarding administration routes. LSW was delivered through two approaches: combined oral-external application (MD = −1.19, 95% CI (−1.72, −0.67), *p* < 0.00001, I^2^ = 77%) and external administration (MD = −1.11, 95% CI (−1.77, −0.45), *p* = 0.001, I^2^ = 71%) (Figure 4). The results demonstrated a statistically significant difference between these two delivery methods. Subgroup analysis was conducted according to the course of treatment. ① ≤ 7 days: experimental group was lower than control group, and the difference was statistically significant (MD = −1.49, 95% CI (−2.31, −0.66), *p* = 0.02, I^2^ = 68%). ② > 8 days: experimental group was lower than control group, and the difference was statistically significant (MD = −1.17, 95% CI (−1.68, −0.66), *p* = 0.03, I^2^ = 71%). ③ NS: experimental group was lower than control group, and the difference was statistically significant (MD = −0.67, 95% CI (−1.68, 0.33), *p* = 0.01, I^2^ = 85%) (Figure 4). Significant heterogeneity persisted despite subgroup analyses based on administration routes and treatment duration, suggesting that other unconsidered factors (e.g., intervention dose, control drug type, variability in conventional treatments) may influence LSW outcomes.

##### 3.4.1.2 Time to scab formation

**FIGURE 5:**
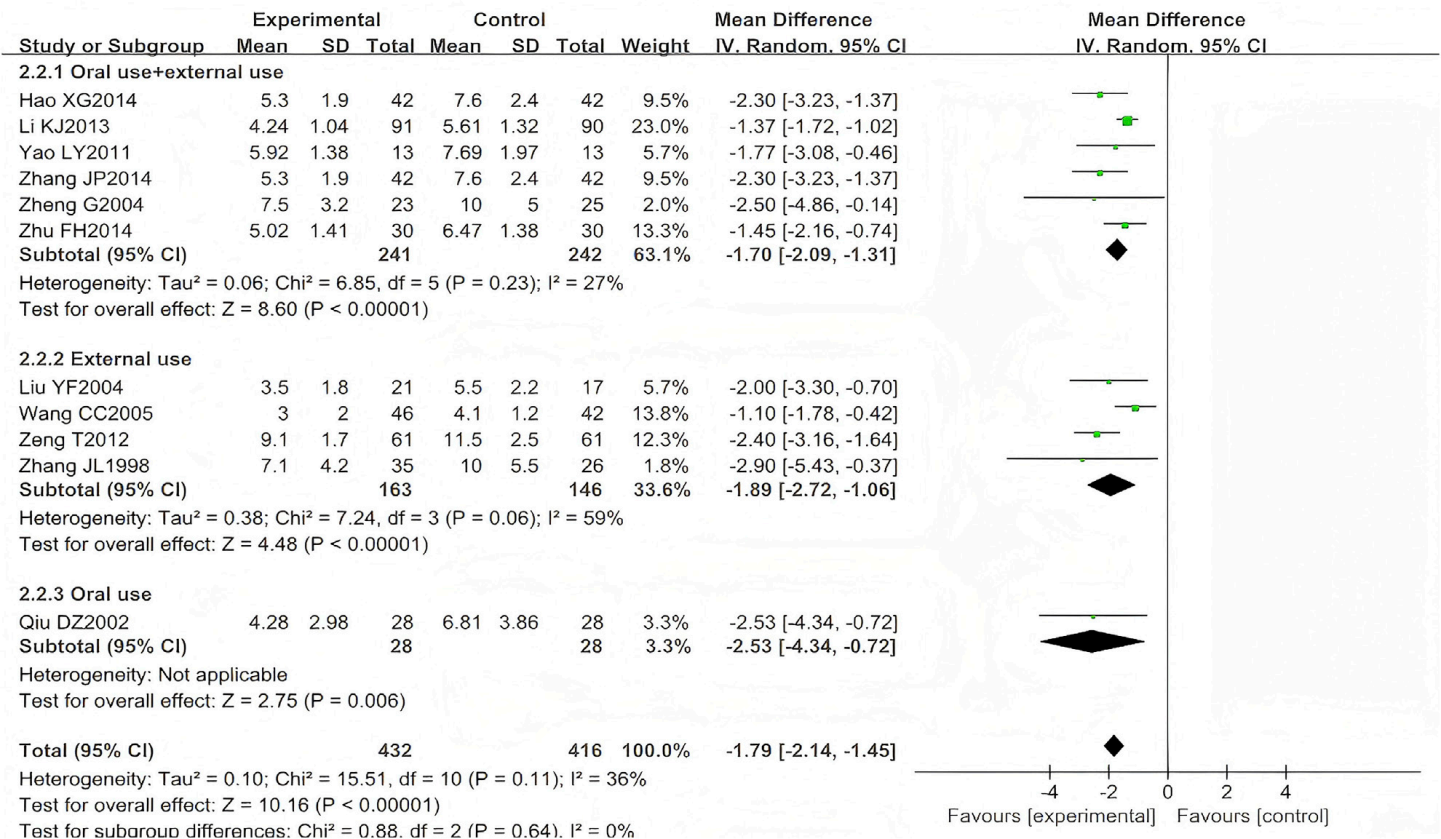
Forest plots with random-effects model for time to scab formation.

A total of 13 studies (n = 968) reported the time to scab formation, among which 10 focused on HZ (n = 764) (Zhang, 1998; Qiu, 2002; Liu, 2004; Wang, 2005; Yao and Sun, 2011; Zeng et al., 2012; Li et al., 2013; Hao, 2014; Zhu, 2014) and one on PHN (n = 84) (Zhang, 2014). Two trials (n = 120) were excluded from the meta-analysis due to unreported standard deviation (SD) for this outcome measure (Dong et al., 2015; Yin and Liang, 2006). To specifically examine the association between time to scab formation and administration routes, this study performed separate meta-analyses, restricting pooled analyses to HZ studies. Scab formation occurred earlier in patients treated with LSW plus conventional therapy than those receiving conventional therapy alone (MD = −1.79, 95% CI (−2.14, −1.45), *p* < 0.00001, I^2^ = 36%) with moderate heterogeneity (Figure 5). The difference was statistically significant. Moreover, following two exclusions of studies with high risk of bias (Yao and Sun, 2011; Zheng, 2004), the direction of the final pooled effect size remained consistent.

##### 3.4.1.3 VAS

A single trial (Dong et al., 2015) provided post-treatment VAS pain score data for PHN. The baseline VAS scores showed no significant intergroup difference (6.47 ± 1.52) in the treatment group vs. the control group (6.19 ± 1.73). The pain score at end of treatment was relatively lower in those receiving LSW with conventional treatment compared to receiving conventional treatment alone.

##### 3.4.1.4 Incidence of PHN

**FIGURE 6:**
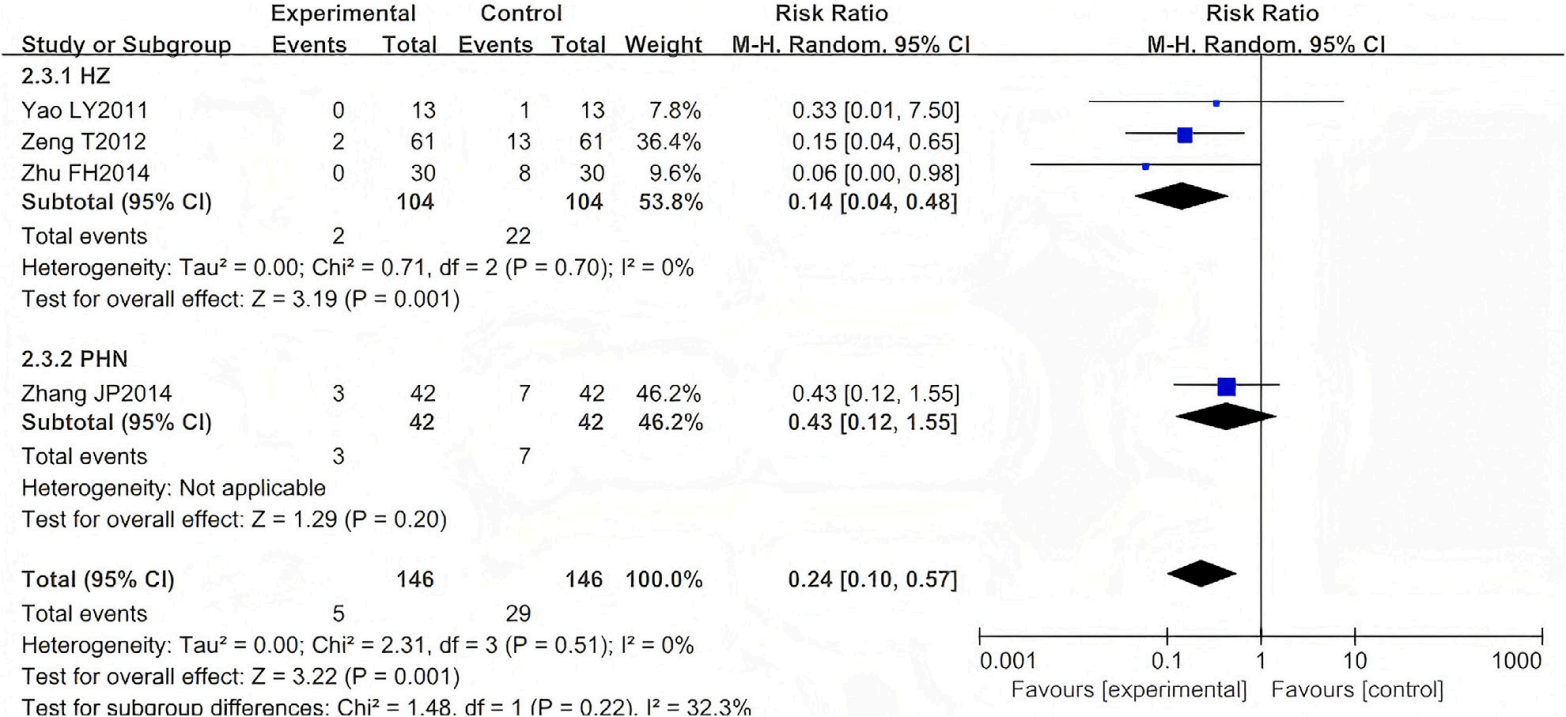
Forest map comparing incidence of postherpetic neuralgia.

Five RCTs were included (Zhang, 1999; Yao and Sun, 2011; Zeng et al., 2012; Zhu, 2014; Zhang, 2014), comprising 376 cases. One study (Zhang, 1999) was excluded from our meta-analysis as both intervention and control groups reported zero occurrence of PHN events—a standard practice when null outcomes occur across arms in OR/RR analyses. The results indicated that the LSW plus conventional treatment group had a statistically significant lower incidence of PHN than the control group (RR = 0.24, 95% CI (0.10, 0.57), *p* = 0.001, I^2^ = 0%) (Figure 6). Heterogeneity tests revealed that the studies were homogeneous (I^2^ = 0%). The direction of the pooled effect estimate did not alter following the sensitivity analysis. One study was deemed to be at high risk of bias (Yao and Sun, 2011).

#### 3.4.2 Secondary outcomes

##### 3.4.2.1 Scab shedding time

**FIGURE 7:**
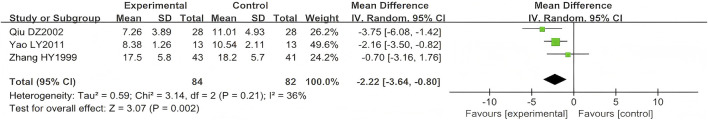
Forest plots with random-effects model for scab shedding time.

Three studies (n = 166) reported on scab shedding time (Qiu, 2002; Yao and Sun, 2011; Zhang, 1999). Their results showed that LSW combined with conventional pharmacotherapy was associated with a significant reduction in time of scab detachment (MD = −2.22, 95% CI (−3.64, −0.80), *p* = 0.002, I^2^ = 36) with moderate heterogeneity (Figure 7). The direction of the effect size remained unchanged, which excluded a high risk-of-bias study (Yao and Sun, 2011), indicating the robustness of the primary finding.

##### 3.4.2.2 Time to pain resolution

**FIGURE 8:**
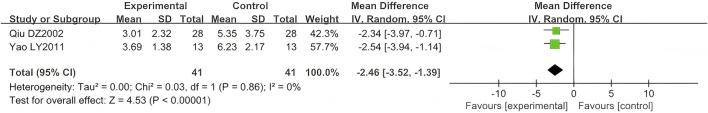
Forest plots with random-effects model for time to pain resolution.

Three studies (n = 162) reported time to the resolution of pain (Yao and Sun, 2011; Qiu, 2002; Dong et al., 2015). One study (n = 80) did not specify the SD (Dong et al., 2015), and data were excluded from this outcome. The comparison results are shown in Figure 8. As a continuous variable, significant differences were also observed in the reduction of time to pain resolution (MD = −2.46, 95% CI (−3.52, −1.39), *p* < 0.00001, I^2^ = 0%).

##### 3.4.2.3 Duration of pain persistence

**FIGURE 9:**
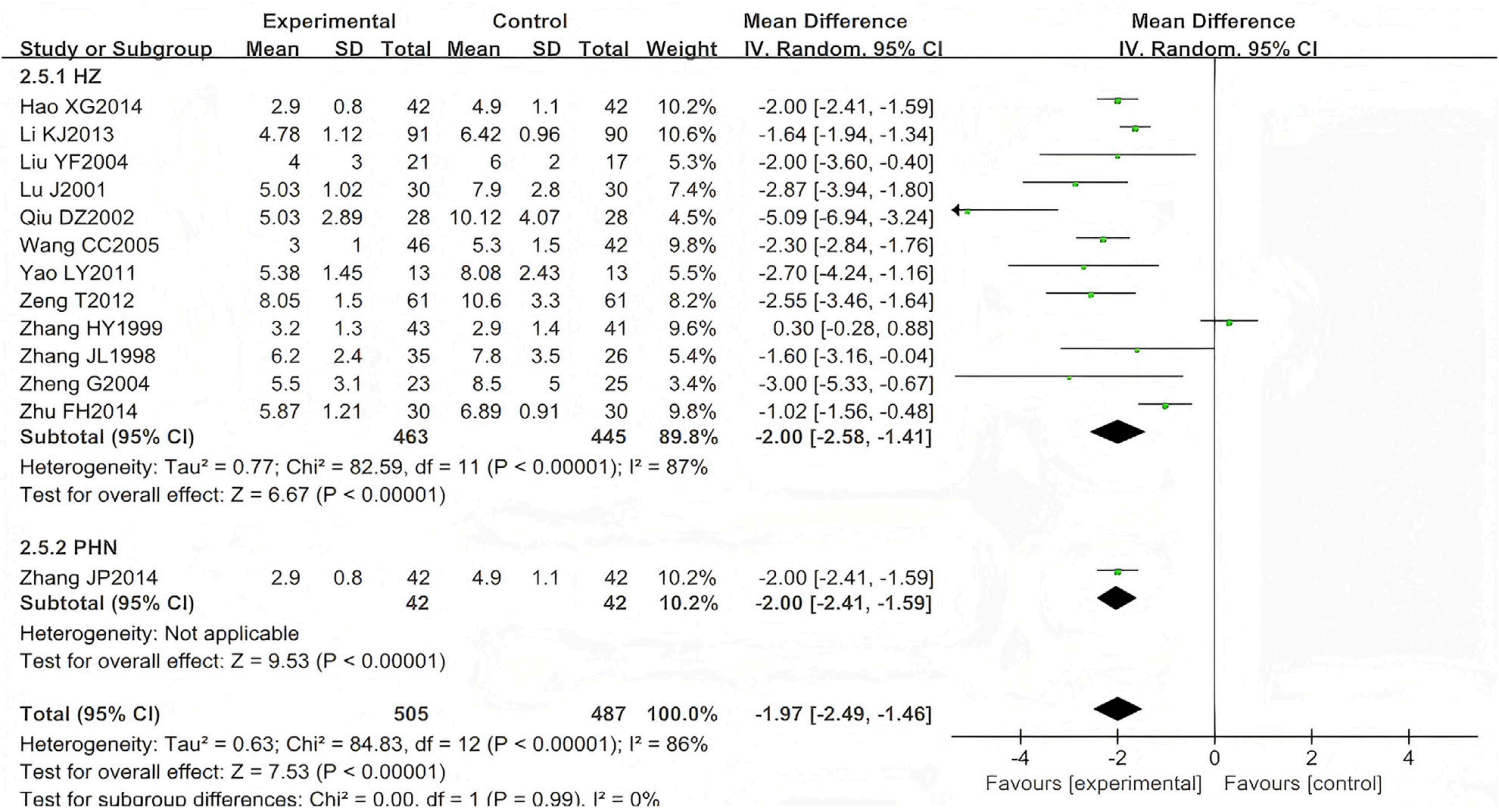
Forest plots with random-effects model for duration of pain persistence.

A total of 17 eligible RCTs (n = 1,156) were identified for inclusion here (Zhang, 1998; Zhang,1999; Lu, 2001; He, 2002; Liu, 2004; Zheng, 2004; Wang, 2005; Fan, 2005; Yin and Liang, 2006; Zhan et al., 2008; Yao and Sun, 2011; Zeng et al., 2012; Li et al., 2013; Hao, 2014; Zhu, 2014; Zhang, 2014; Qiu, 2002). Four (n = 164) (He, 2002; Fan, 2005; Yin and Liang, 2006; Zhan et al., 2008) were excluded from the meta-analysis due to unreported temporal measurement parameters (time-specific standard deviations), consistent with *Cochrane Handbook* guidelines on incomplete continuous data. The results showed that the experimental group was better than the control group in shortening the duration of pain persistence, and the difference was statistically significant (MD = −1.97, 95% CI (−2.49, −1.46), *p* < 0.00001, I^2^ = 86%) (Figure 9). After excluding three studies (Zhang, 1999; Qiu, 2002; Zhu, 2014), the heterogeneity was substantially reduced (I^2^ = 26%, *p* < 0.00001), suggesting that these studies were the primary source of the observed heterogeneity.

##### 3.4.2.4 The efficacy of PHN

**FIGURE 10:**
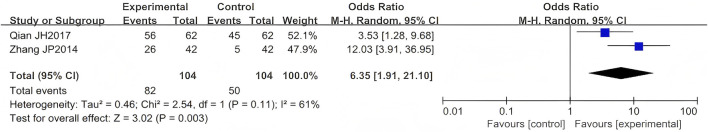
Forest map comparing efficacy of postherpetic neuralgia.

Two RCTs evaluated LSW as adjunctive therapy for PHN. Meta-analysis demonstrated a statistically significant improvement in efficacy for LSW combined with conventional treatment compared to conventional therapy alone (OR = 6.35, 95% CI (1.91, 21.10), *p* = 0.003, I^2^ = 61%) (Figure 10) (Qian and Lu, 2017; Zhang, 2014), indicating a strong therapeutic benefit of LSW in PHN management. This meta-analysis revealed substantial heterogeneity (I^2^ = 61%), suggesting significant variation across the included studies.

##### 3.4.2.5 Adverse drug reactions

**FIGURE 11:**
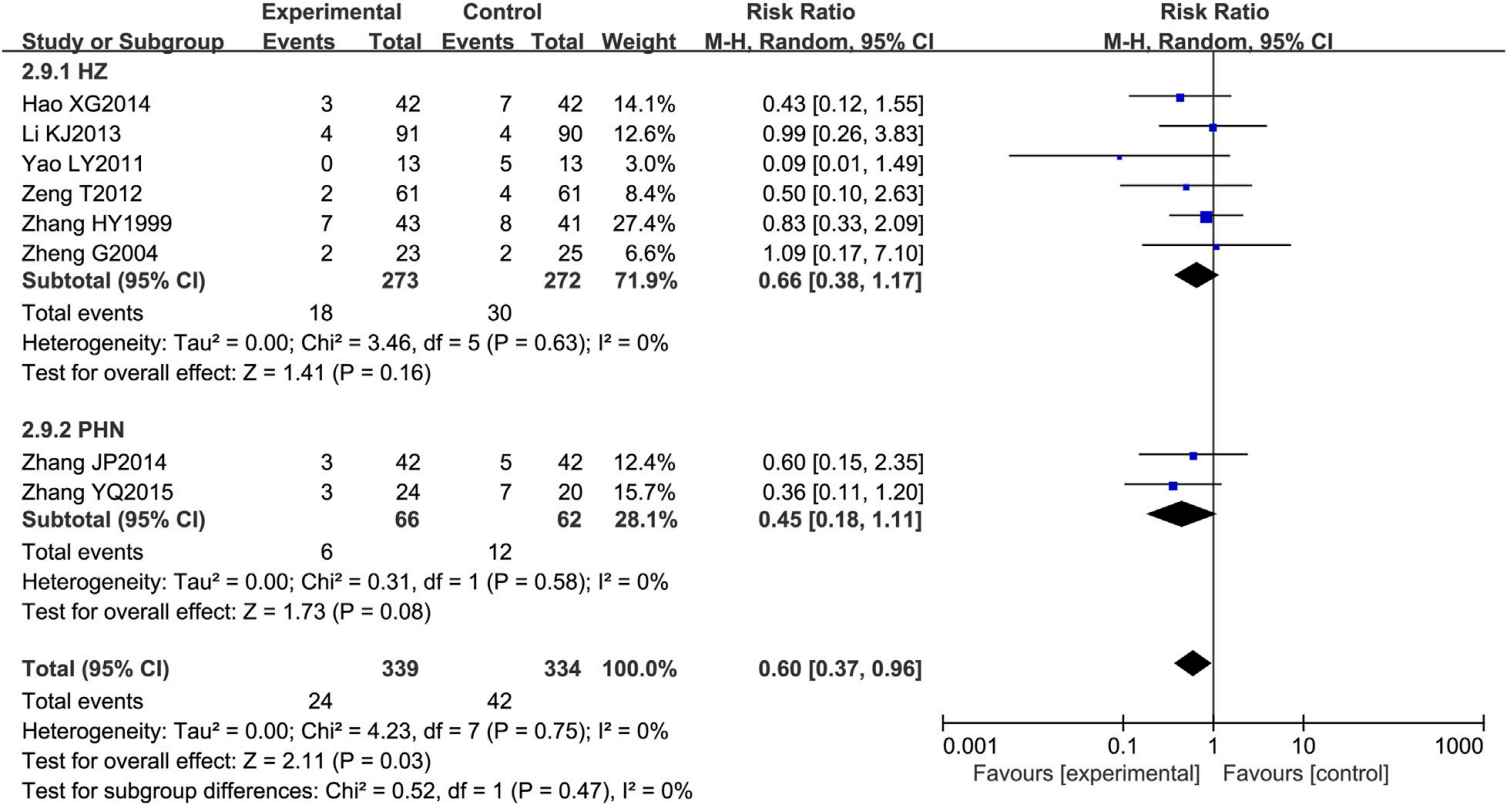
Forest plots with random-effects model of adverse events.

Among the 21 included RCTs, 8 mentioned adverse drug reactions (ADRs). The most commonly reported ADRs were gastrointestinal disturbances, including nausea, diarrhea, abdominal pain, and bloating (reported in five studies) (Zhang, 1999; Zheng, 2004; Yao and Sun, 2011; Li et al., 2013; Zhang et al., 2015), followed by central nervous system effects such as dizziness and drowsiness (three studies) (Zhang, 1999; Zheng, 2004; Zhang et al., 2015). Other notable ADRs included cutaneous pruritus (one study) (Zhang, 1999) and unspecified adverse drug reactions (five studies) (Li et al., 2013; Hao, 2014; Zeng et al., 2012). The incidence of adverse effects associated with LSW plus conventional treatment was low (RR = 0.60, 95% CI (0.37, 0.96), *p* = 0.03, I^2^ = 0%), with no severe adverse reactions reported (Figure 11). There was no substantial heterogeneity among these eight trials. The details of adverse events are presented in Table 3.

**TABLE 3:** Adverse reactions and safety indicators.

Study ID	Adverse events	*p*-value	Safety indicator
Yao and Sun (2011)	Mild gastrointestinal reactions (T, 0 case; C, 5 cases)	NR	NR
Hao (2014)	No statistical significance between two groups (T, 3 cases; C, 5 cases)	*p* > 0.05	NR
Li et al. (2013)	Diarrhea (T, 1 case; C, 2 cases); abdominal pain and diarrhea (T, 2 cases; C, 0 case)	NR	NR
Zeng et al. (2012)	Mild headache (T, 2 cases; C, 4 cases); no statistical significance between two groups	*p* > 0.05	NR
Zhang (1999)	Dizziness (T, 2 cases; C, 0 case), gastrointestinal reactions (T, 3 cases; C, 8 cases), application site pruritus (T, 2 cases; C, 0 case)	NR	NR
Zheng (2004)	Nausea (T, 0 case; C, 2 cases); dizziness and drowsiness (T, 2 cases; C, 0 case, resolved after discontinuation). No serious adverse effect reported	NR	NR
Zhang (2014)	(T, 3 cases; C, 5 cases)	*p* > 0.05	NR
Zhang (2015)	Dizziness (T, 1 case; C, 2 cases), nausea and abdominal distension (T, 1 case; C, 0 case) Somnolence and fatigue (T, 1 case; C, 3 cases), others (T, 0 case; C, 2 cases)	*p* < 0.05	NR

Abbreviation: T, treatment group; C, control group; NR, not reported.

### 3.5 Publication bias

**FIGURE 12:**
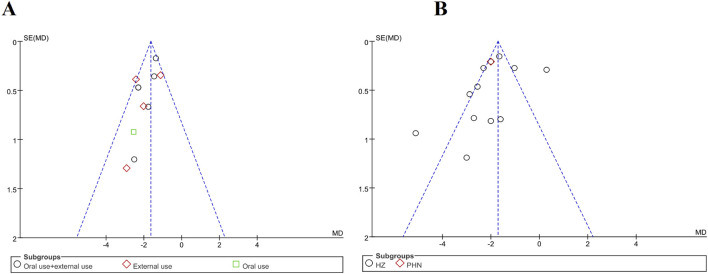
Funnel plot. **(A)** Time to scab formation. **(B)** Duration of pain persistence.

There were 11 trials that evaluated time to scab formation and 12 that evaluated the duration of pain persistence in meta-analysis. Funnel plots were generated for both outcomes to examine potential publication bias (Figures 12A,B). The asymmetry was not pronounced, and no publication bias was observed. Notably, none of the trials reported methods for sample size estimation, and all eligible trials originated from Chinese publications. In Figure 12B, three studies fall outside inverted funnel boundaries. Further details of the funnel plot analysis are provided in Figure 12.

### 3.6 Evidence quality assessment

The GRADE approach was used to assess the quality of evidence for seven outcomes across all included studies. The evidence quality was low for vesicle cessation time and the duration of pain persistence, high for time to scab formation, and moderate for other outcomes. With results from the seven aspects of the “bias risk evaluation” tool mentioned above, the main reasons for the result of “low quality” of the comparison evaluation are as follows: a. most information is from studies at low or unclear risk of bias; b. the proportion of information from studies at high risk of bias is sufficient to affect the interpretation of results; c. there was substantial heterogeneity, as noted by 60 < I^2^<90% (downgraded by one level for inconsistency); d. there was substantial heterogeneity, as noted by I^2^>75% (downgraded by one level for inconsistency); and e. potential limitations are likely to lower confidence in the estimate of effect. The GRADE evidence profiles of outcomes are presented in Supplementary Material B.

### 3.7 ConPhyMP assessment

The 21 included RCTs were evaluated against the ConPhyMP reporting guidelines (Supplementary Material C). Upon reviewing the titles and abstracts, it was found that all studies fully complied with Item 1 by providing a clear and concise title, including an informative, balanced summary. Conversely, several items were uniformly absent across all literature: none of the studies provided details for Item 2 (description of the botanical drug and taxonomic authentication), Item 3 (description of the extract and extraction process), Item 4 (documentation of the legal basis for collection and processing), or Item 5 (product characteristics).

The assessment of included studies confirmed that all botanical drugs were documented in national pharmacopoeias, fulfilling Item 1. However, nine ConPhyMP criteria were consistently unreported across the literature. These omissions included the following: the description of active ingredients or defined analytical markers (Item 2a*); required monograph-based analysis for non-certified extracts (Item 2b*); provision of manufacturer and assay certificates (Item 2c*); application of triple chemical fingerprinting (Item 2a^#^); quantification of at least two labeled compounds (Item 2b^#^); description of single chemical fingerprinting with varied detection parameters (Item 3a); specification for quantified markers (Item 3b); use of reference standards (Item 4); and comparative analysis of different extracts or batches (Item 5).

## 4 Discussion

The findings of this systematic review and meta-analysis suggest that LSW plus biomedicine for HZ and PHN are significantly more effective than conventional treatment alone, although this conclusion is based on a limited number of reported studies. The pooled analysis revealed that LSW as an add-on therapy to conventional therapy appeared to reduce the time to shorten the vesicle cessation time, time to scab formation, scab shedding time, time to pain resolution, and the duration of pain persistence compared to conventional treatment alone. In addition, adjuvant treatment with LSW could also decrease the incidence of PHN, improve the efficacy of PHN, and lower the occurrence of adverse reactions. No serious adverse drug events were reported. Overall, LSW is a promising adjunctive therapy, but its significant benefits must be weighed against the increased risk of minor adverse events, highlighting the need for clinical monitoring.

Herpes zoster (HZ) is characterized by three cardinal features: (1) acute onset; (2) blister developing in a zonal region innervated from the ganglion; (3) neuropathic pain (Maeda et al., 2019). Our meta-analysis of 21 RCTs (n = 1,478) demonstrates that LSW combined with conventional therapy significantly accelerates all three key healing phases—vesicle cessation, scab formation, and complete scab shedding—compared to conventional treatment alone. The observed clinical benefits of LSW find support in both traditional Chinese medicine (TCM) theory and contemporary pharmacological research. In TCM, HZ results from liver fire excess and spleen dampness retention, leading to damp-heat obstruction of meridians requiring treatment focused on heat-clearing, detoxification, dampness-draining, and pain relief (Miao and Liu, 2011). TCM attributes HZ to liver-fire and spleen-dampness pathogenesis, which LSW addresses through its targeted combination of heat-clearing *Calculus bovis*, detoxifying realgar, and pain-relieving *Moschus*. Recently, LSW exhibited significant antiviral activity by suppressing influenza virus replication, mediated through the downregulation of virus-induced inflammatory cytokines via modulation of the TLR4/NF-κB signaling pathway (Ma et al., 2020).

Acute zoster-associated pain affecting most HZ patients often responds inadequately to conventional non-opioid analgesics (Iseki et al., 2024). This neuropathic pain profoundly impairs quality of life, manifesting as sleep disturbance, social dysfunction, and elevated depression risk (Liu et al., 2023). In our meta-analysis, the LSW-plus-conventional therapy group demonstrated superior pain reduction versus conventional treatment alone, despite comparable baseline scores (Zhang et al., 2015). Notably, this aligns with a prior trial where LSW without adjunct analgesics outperformed ibuprofen-based therapy (Yao and Sun, 2011), suggesting LSW’s intrinsic analgesic properties. These results suggest that LSW may work in two ways: fighting the virus while also reducing nerve pain. This could make it more effective than standard antiviral treatments alone.

Postherpetic neuralgia (PHN) develops in 9%–34% of HZ patients, showing exponential age-dependent risk progression, yet many experience suboptimal treatment responses or intolerable adverse effects (Liu et al., 2020). Our meta-analysis demonstrates that LSW adjunct therapy can reduce PHN incidence and improve treatment efficacy. Clinical observations demonstrate that antiviral agents alone fail to adequately control PHN symptoms, with frequent pain recurrence upon discontinuation (Qian and Lu, 2017). In contrast, the topical application of LSW effectively manages pain symptoms. The use of oral and topical LSW as an adjunctive therapy shows favorable safety and efficacy profiles whilst providing sustained PHN symptom control (Xuerui et al., 2021). These findings support its clinical adoption for comprehensive HZ management.

It was well-documented that realgar (*Xionghuang*), a primary ingredient in *Liushen* pill, contains arsenic, a substance associated with a known risk of toxic effects with chronic or improper use (Shixia et al., 2016; Shi-Xia et al., 2011). In our meta-analysis, the pooled results from the available studies suggested a low incidence of adverse events associated with its short-term use in the included clinical contexts. The most frequently reported adverse events were mild gastrointestinal disturbances (nausea, diarrhea, abdominal pain). Central nervous system effects such as dizziness and drowsiness (Zhang, 1999; Zheng, 2004; Yao and Sun, 2011; Li et al., 2013; Zhang et al., 2015) are the most common adverse events with LSW and may result from As_4_S4 (Liu et al., 2008). However, we fully acknowledge the limitations of this safety evidence. The included studies were primarily focused on efficacy and lacked systematic assessment and reporting of specific adverse events, laboratory parameters related to toxicity (e.g., heavy metal levels, liver and renal function), and potential drug interactions. Therefore, it needs to be reevaluated for safety. The study of state on the heavy metal in LSW and its interaction with organic constituents confirmed that the synthesized cysteine-arsenic (III) complex is significantly less toxic than As_2_O_3_, indicating that cysteine effectively antagonizes the toxicity of arsenic (Xiuping et al., 2003). By establishing an inductively coupled plasma mass spectrometry (ICP-MS) method for the determination of total arsenic in blood and a high-performance liquid chromatography (HPLC)–ICP-MS method for arsenic speciation, research findings demonstrate that the arsenic in *Liushen* pill has a shorter mean residence time and lower tissue retention than that in pure realgar, being predominantly transformed into the less toxic dimethylarsinic acid (DMA) (Qizheng et al., 2016; Qing-Li et al., 2011). Thus, the other herbal components in the formula likely reduce arsenic toxicity, potentially by facilitating its detoxification and elimination (Qian, 2007; Qizheng et al., 2016). Furthermore, Jing et al. (2018) reported that the inclusion of *Calculus bovis* in LSW alleviates the cardiotoxicity induced by *Venenum bufonis*, as the bilirubin and taurine in *calculus bovis* inhibit intracellular sodium elevation and subsequent calcium overload in cardiomyocytes, demonstrating that the formula’s compatibility reduces the toxicity of both realgar and *Venenum bufonis*.

The methodological limitations of the included studies, as identified by the RoB 2.0 assessment, warrant careful consideration. “Some concerns” regarding blinding in several trials are non-trivial. For instance, our primary outcome of the “incidence of postherpetic neuralgia” and secondary outcomes like “pain relief” are highly subjective. The inability to ensure the effective blinding of patients and caregivers in these trials may have introduced performance and detection bias, potentially leading to an over-optimistic estimate of the intervention’s benefit. Therefore, the magnitude of the treatment effect for these subjective outcomes should be interpreted with caution.

## 5 Limitations

The present study has several limitations. First, as all included studies were conducted in China, the findings may be influenced by specific populations (such as TCM diagnosis and treatment habits) and medical environments, which may affect the generalizability of the conclusions. Furthermore, variability in conventional treatments was probably a key source of heterogeneity among the studies. To enhance the universality of the findings, it is recommended that verification studies be carried out in multiple regions in the future. Second, the outcomes of skin lesions in HZ patients are commonly assessed in terms of incrustation and decrustation time in clinical settings. These outcomes mainly depend on the physician’s discretion, which may easily result in bias detection. Third, while this meta-analysis suggests potential benefits of *Liushen* pill combination therapy, it must be acknowledged that the evidence has limitations in its persuasiveness. As highlighted by the GRADE approach, the overall certainty of the evidence for outcomes was rated low to moderate. This was primarily because the risk of bias in some included studies was the large number of poorly or non-reported aspects (blinding allocation concealment). This high prevalence of unclear or high risk of bias in critical domains substantially compromises the reliability of the efficacy results. Furthermore, it should be emphasized that the safety analysis was based on a small overall sample size. Existing fundamental research on HZ, as well as large-scale epidemiological and prevention data, remain limited. Consequently, this study inevitably has certain limitations.

## 6 Conclusion

This review demonstrates that LSW combined with conventional biomedical treatment offers clinically meaningful benefits in herpes zoster management, including accelerated lesion healing, reduced duration of pain persistence, reduced postherpetic neuralgia incidence, and favorable safety profiles. However, the current evidence base is constrained by methodological shortcomings and heterogeneity, precluding definitive conclusions. The findings of positive effects must be interpreted with extreme caution and should be viewed as hypothesis-generating and reflecting a promising signal of potential benefit rather than providing conclusive proof. Clinicians should consider integrating LSW into individualized treatment plans while accounting for the current evidence limitations and patient preferences. The primary value of this analysis lies in synthesizing the existing—albeit methodologically limited—literature and in highlighting the urgent need for more rigorously designed and transparently reported future trials.

## Data Availability

The original contributions presented in the study are included in the article/Supplementary Material; further inquiries can be directed to the corresponding authors.

## Abbreviations

LSW, *Liu Shen wan*; CCPP, commercial Chinese polyherbal preparation; TCM, traditional Chinese medicine; HZ, herpes zoster; PHN, postherpetic neuralgia; VZV, varicella-zoster virus; RCTs, randomized controlled trials; RR, risk ratio; OR, odds ratio; MD, mean difference; SMD, standardized mean difference; CI, confidence intervals; GRADE, Grades of Recommendations Assessment, Development, and Evaluation; VAS, visual analog scale; NS, not specified; C, control group; I, intervention group; PDS, potassium dehydroandrographolide succinate for injection; BCG-PSN, BCG polysaccharide and nucleic acid preparation; SPSS, stroke-physiological saline solution; VB, vitamin B; Poly (I:C), polyinosinic–polycytidylic acid injection; DS, diclofenac sodium; GS, glucose injection.
