# Supplementary material for: Efficacy and safety of the commercial Chinese polyherbal preparation Liu Shen Wan as an adjunctive treatment for herpes zoster and postherpetic neuralgia: a systematic review and meta-analysis
Source: Front Pharmacol. 2025 Nov 28;16:1698753. doi: 10.3389/fphar.2025.1698753 (PMC12698603; doi:10.3389/fphar.2025.1698753)
Supplement: Supplementary file 1 [file Supplementaryfile1.docx]

Supplementary A

**1. PUBMED (Search date: June 28, 2024(updated on April 6, 2025))**

Search terms ：

#1 herpes zoster[MeSH Terms] 13623

#2 shingles[MeSH Terms] 13623

#3 (((Liu-Shen-Wan[Title/Abstract]) OR (liu-shen-wan[Title/Abstract])) OR (liushenwan[Title/Abstract])) OR (LiuShenWan[Title/Abstract]) 16

#4 (herpes zoster[Title/Abstract]) OR (shingles[Title/Abstract]) 13081

#5 #1 OR #2

#6 #4 OR #5 18247

#7 #3 AND #6

**2. EMBASE (Search date: June 28, 2024(updated on April 6, 2025))**

Search terms ：'LiuShenWan'/exp OR LiuShenWan OR 'moxa'/exp OR moxa AND ('herpes zoster'/exp OR herpes zoster OR 'postherpetic neuralgia'/exp OR postherpetic neuralgia OR 'zona'/exp OR zona OR 'zoster'/exp OR zoster OR 'shingles'/exp OR shingles) AND (randomized OR trial)

**3. The Cochrane Library (Search date: June 28, 2024(updated on April 6, 2025))**

Search terms ：1. herpes zoster*:ti,ab OR postherpetic neuralgia*:ti,ab OR zona*:ti,ab OR zoster*:ti,ab OR shingles*:ti,ab OR zoster herpes*:ti,ab

2. MeSH descriptor: [Herpes Zoster] explode all trees

3. MeSH descriptor: [Neuralgia, Postherpetic] explode all trees

4. #1 OR #2 OR #3

5. MeSH descriptor: [LiuShenWan] explode all trees

6. LiuShenWan:ti,ab OR moxa:ti,ab

7. #5 OR #6

8. #4 AND #7 (limited to “trials”)

**4. ClinicalTrials.gov (Search date: June 28, 2024(updated on April 6, 2025))**

Search terms：LiuShenWan

**5. Web of science (Search date: June 28, 2024(updated on April 6, 2025))**

Search terms: (LiuShenWan*[Title/Abstract] OR LiuShenWan*[TOPIC] OR moxabustion*[TOPIC] OR moxa*[TOPIC]) AND (herpes zoster*[Title/Abstract] OR postherpetic neuralgia*[Title/Abstract] OR herpes zoster*[TOPIC] OR postherpetic neuralgia*[TOPIC] OR zona*[TOPIC] OR zoster*[TOPIC] OR shingles*[TOPIC] OR zoster herpes*[TOPIC])

**6. China National Knowledge Infrastructure (CNKI) (Search date: June 28, 2024(updated on April 6, 2025))**

Search terms: (SU = 六神丸) AND ( SU ='带状疱疹' OR SU = '蛇串疮' OR SU = '蛇缠腰' OR SU = '缠腰火丹' OR SU = '火带疮' OR SU = '火丹' OR SU = '带状性疱疹' OR SU = '蜘蛛疮' OR SU = '蛇盘疮' OR SU = '蛇丹' OR SU = '带状疱疹后遗神经痛') AND ( FT = '随机')

**7. WanfangData (Search date: June 28, 2024(updated on April 6, 2025))**

Search terms:题名: 六神丸 AND 题名: ("带状疱疹"OR"蛇串疮"OR"缠腰火丹"OR"火带疮"OR"火丹"OR"带状性疱疹"OR"蜘蛛疮"OR"蛇盘疮"OR"蛇丹"OR"带状疱疹后遗神经痛") AND 摘要: 随机

**8. VIP Database for Chinese Technical Periodicals (VIP) (Search date: June 28, 2024(updated on April 6, 2025))**

Search terms: T=六神丸 AND T=(带状疱疹 OR 蛇串疮 OR 缠腰火丹 OR 火带疮 OR 火丹 OR 带状性疱疹 OR 蜘蛛疮 OR 蛇盘疮 OR 蛇丹 OR 带状疱疹后遗神经痛) AND U=随机

1. **China Biomedical Network Information (Search date: June 28, 2024(updated on April 6, 2025))**

Search terms: 六神丸[主题词] AND (带状疱疹[主题词] OR 带状疱疹后遗神经痛[主题词] OR 带状疱疹[全部字段] OR 带状疱疹后遗神经痛[全部字段] OR蛇串疮[全部字段] OR 缠腰火丹[全部字段] OR 火带疮[全部字段] OR 火丹[全部字段] OR 带状性疱疹[全部字段] OR 蜘蛛疮[全部字段] OR 蛇盘疮[全部字段] OR 蛇丹[全部字段] OR 带状疱疹后遗神经痛[全部字段]) AND 随机[全部字段]
