# Supplementary material for: Efficacy and safety of the commercial Chinese polyherbal preparation Liu Shen Wan as an adjunctive treatment for herpes zoster and postherpetic neuralgia: a systematic review and meta-analysis
Source: Front Pharmacol. 2025 Nov 28;16:1698753. doi: 10.3389/fphar.2025.1698753 (PMC12698603; doi:10.3389/fphar.2025.1698753)
Supplement: Supplementary file 3 [file Supplementaryfile2.docx]

Supplementary B

| **LSW plus conventional therapy compared to the conventional therapy for herpes zoster and postherpetic neuralgia**  **Bibliography:** | | | | | | | | | | | |
| --- | --- | --- | --- | --- | --- | --- | --- | --- | --- | --- | --- |
| **Certainty assessment** | | | | | | | **Summary of findings** | | | | |
| **Participants (studies) Follow-up** | **Risk of bias** | **Inconsistency** | **Indirectness** | **Imprecision** | **Publication bias** | **Overall certainty of evidence** | **Study event rates (%)** | | **Relative effect (95% CI)** | **Anticipated absolute effects** | |
|  |  |  |  |  |  |  | **With the conventional therapy** | **With LSW plus conventional therapy** |  | **Risk with the conventional therapy** | **Risk difference with LSW plus conventional therapy** |
| **Vesicle Cessation Time** | | | | | | | | | | | |
| 789 (9 RCTs) | serious^a,b^ | serious^c^ | not serious | not serious | none | ⨁⨁◯◯ Low^a,b,c^ | 390 | 399 | - | 390 | MD **1.17 lower** (1.54 lower to 0.8 lower) |
| **Time to scab formation** | | | | | | | | | | | |
| 848 (11 RCTs) | not serious | not serious | not serious | not serious | none | ⨁⨁⨁⨁ High | 416 | 432 | - | 416 | MD **1.79 lower** (2.14 lower to 1.45 lower) |
| **Scab shedding time** | | | | | | | | | | | |
| 166 (3 RCTs) | serious^d^ | not serious | not serious | not serious | none | ⨁⨁⨁◯ Moderate^d^ | 82 | 84 | - | 82 | MD **2.22 lower** (3.64 lower to 0.8 lower) |
| **Duration of pain persistence** | | | | | | | | | | | |
| 992 (13 RCTs) | not serious | very serious^e^ | not serious | not serious | none | ⨁⨁◯◯ Low^e^ | 487 | 505 | - | 487 | MD **1.97 lower** (2.49 lower to 1.46 lower) |
| **Time to pain resolution** | | | | | | | | | | | |
| 82 (2 RCTs) | serious^d^ | not serious | not serious | not serious | none | ⨁⨁⨁◯ Moderate^d^ | 41 | 41 | - | 41 | MD **2.46 lower** (3.52 lower to 1.39 lower) |
| **The occurrence of PHN** | | | | | | | | | | | |
| 292 (5 RCTs) | serious^a,b^ | not serious | not serious | not serious | none | ⨁⨁⨁◯ Moderate^a,b^ | 29/146 (19.9%) | 5/146 (3.4%) | **RR 0.24** (0.10 to 0.57) | 29/146 (19.9%) | **151 fewer per 1,000** (from 179 fewer to 85 fewer) |
| **The efficacy of PHN** | | | | | | | | | | | |
| 208 (2 RCTs) | not serious | serious^c^ | not serious | not serious | none | ⨁⨁⨁◯ Moderate^c^ | 50/104 (48.1%) | 82/104 (78.8%) | **OR 6.35** (1.91 to 21.10) | 50/104 (48.1%) | **374 more per 1,000** (from 158 more to 471 more) |
| **Adverse drug reactions** | | | | | | | | | | | |
| 747 (9 RCTs) | serious^d^ | not serious | not serious | not serious | none | ⨁⨁⨁◯ Moderate^d^ | 46/364 (12.6%) | 26/383 (6.8%) | **RR 0.57** (0.36 to 0.90) | 46/364 (12.6%) | **54 fewer per 1,000** (from 81 fewer to 13 fewer) |

**CI:** confidence interval; **MD:** mean difference; **OR:** odds ratio; **RR:** risk ratio

**Explanations**

a. Most information is from studies at low or unclear risk of bias.

b. Potential limitations are likely to lower confidence in the estimate of effect.

c. There was substantial heterogeneity as noted by 60＜I2＜90% (Downgraded by one level for inconsistency)

d. The proportion of information from studies at high risk of bias is sufficient to affect the interpretation of results.

e. There was substantial heterogeneity as noted by I2＞75% (Downgraded by one level for inconsistency)
